# A Lard and Soybean Oil Mixture Alleviates Low-Fat–High-Carbohydrate Diet-Induced Nonalcoholic Fatty Liver Disease in Mice

**DOI:** 10.3390/nu14030560

**Published:** 2022-01-27

**Authors:** Sisi Yan, Sha Liu, Jianyu Qu, Xiaowen Li, Jiahao Hu, Linyu Zhang, Xiangyan Liu, Xin Li, Xianglin Wang, Lixin Wen, Ji Wang

**Affiliations:** 1 Hunan Engineering Research Center of Livestock and Poultry Health Care, Colleges of Veterinary Medicine, Hunan Agricultural University, Changsha 410128, China; yss06091993@163.com (S.Y.); ls100585@stu.hunau.edu.cn (S.L.); QJY@stu.hunau.edu.cn (J.Q.); lxw0630@stu.hunau.edu.cn (X.L.); hujiahao914@stu.hunau.edu.cn (J.H.); zhanglinyu@stu.hunau.edu.cn (L.Z.); liuxiangyan@stu.hunau.edu.cn (X.L.); lixin0822@stu.hunau.edu.cn (X.L.); wangxianglin0921@163.com (X.W.); 2 National Engineering Laboratory for Deep Process of Rice and Byproducts, College of Food Science and Engineering, Central South University of Forestry and Technology, Changsha 410004, China

**Keywords:** lard, soybean oil, low-fat–high-carbohydrate diet, nonalcoholic fatty liver disease

## Abstract

Dietary habit is highly related to nonalcoholic fatty liver disease (NAFLD). Low-fat–high-carbohydrate (LFHC) diets could induce lean NAFLD in Asians. Previously, we found that a lard and soybean oil mixture reduced fat accumulation with a medium-fat diet; therefore, in this study, we evaluated the effect of a lard and soybean oil mixture (LFHC diet) on NAFLD and its underlying mechanisms. Mice in groups were fed with lard, soybean oil, or a lard and soybean oil mixture—an LFHC diet—separately. Our results showed that mixed oil significantly inhibited serum triglyceride, liver triglyceride, serum free fatty acids (FFAs), and liver FFAs compared with soybean oil or lard, and we found fewer inflammatory cells in mice fed with mixed oil. RNA-seq results indicate that mixed oil reduced FFAs transportation into the liver via decreasing liver fatty acid-binding protein 2 expression, inhibited oxidative phosphorylation via tumor necrosis factor receptor superfamily member 6 downregulation, and alleviated inflammation via downregulating inflammatory cytokine. The liquid chromatography–mass spectrometry results showed that the mixed oil promoted bile acid conjugated with taurine and glycine, thus activating G-protein-coupled bile acid receptor 1 for improved lipids metabolism. In conclusion, the lard and soybean oil mixture alleviated NAFLD.

## 1. Introduction

Nonalcoholic fatty liver disease (NAFLD) results from at least 5% of hepatocytes having steatosis, but not caused by known damage, such as alcohol, viruses, or drugs [1]. NAFLD is considered as a range of liver abnormalities from nonalcoholic fatty liver (NAFL) to nonalcoholic steatohepatitis (NASH), it has a variable course, and it can lead to cirrhosis and liver cancer. At the same time, NASH is a more stringent process with inflammation and hepatocyte damage (steatohepatitis) [2]. According to the available data, NAFLD incidences are highest in the Middle East and South America, with an estimated global prevalence of NAFLD of 25% [3]. NAFLD is the leading cause of chronic liver disease in the United States, affecting between 80 million and 100 million people, of which nearly 25% of cases progress to NASH [4]. The prevalence of NAFLD in Asia is 27.4% higher than that of Europe (23.7%), with an incidence rate of 43.9% in mainland China alone [5]. The NAFLD case number is projected to increase from 200 million in 2016 to 300 million in 2030: a 29.1% increase that presents a growing economic burden [6]. Interestingly, NAFLD typically is accompanied by central obesity, as seen in North Americans and Europeans (~83% of patients); however, a sizable percentage of Asian patients with “lean NASH” have normal body mass index (BMI)—the BMI cutoff for overweight in Asia (BMI > 23) is lower than in North America and Europe (BMI > 25) [7]. It is inferred that this might be due to varying differences in dietary patterns; while high-fat diets are predominant in America and Europe, high-carbohydrate diets are predominant in China. High-carbohydrate diets are thought to be as harmful as a high-fat diets in the development and progression of liver injury in mice [8]. A long-term low-fat–high-carbohydrate diet (LFHC) consumption could induce NAFLD in mice [9], while a high-carbohydrate diet could substantially induce a lower body weight gain [8,9]. Thus, an LFHC diet is considered superior to methionine- and choline-deficient diets for NASH models. The reason for this is that patients with NASH are not choline deficient; therefore, methionine- and choline-deficient diets would only induce a histological appearance similar to NASH—with fat, inflammation, and fibrosis. These underlying defects are not related to human disease pathogenesis [2].

In NAFLD development, the initial step involves liver fat accumulation. Three major mechanisms are associated with excessive fat accumulation, as follows: (1) excessive free fatty acids (FFAs) flux from the adipose tissue to the liver (59%); (2) hepatic de novo lipogenesis (26%); (3) excessive diet lipids and calorie levels (15%) [10]. Hormone-sensitive lipase (HSL) is a speed-limiting enzyme which occurs during triglyceride (TG) hydrolysis to FFAs [11]. De novo lipogenesis is controlled by two transcriptional factors; one is the sterol regulatory element-binding protein 1c [12]. Fatty acid-binding proteins (FABPs) are a family of cytoplasmic proteins that binds long-chain fatty acids and participates in fatty acid uptake and transport [13]. Complex mechanisms are involved in the evolution of simple steatosis to NASH. The accumulation of FFAs in the liver predisposes lipotoxicity and promotes NASH development. Excess FFAs can directly induce oxidative stress and reactive oxygen species (ROS)—increased concentration of ROS induces hepatocytes death by activating specific pathways [14,15]. Excess FFAs causes cellular damage directly, by modifying endoplasmic reticulum and mitochondrial biology and functions, intracellular signaling pathways, and interacting with specific proinflammatory cellular kinases [12]. The “remodeling” of mitochondrial energetics, including β-oxidation, hepatic tricarboxylic acid cycle, and oxidative phosphorylation, play a vital role in the metabolic adaption to FFAs overloads, which are overexpressed in patients with NAFLD [16]. NAFLD does not just affect the liver, it also affects other organs. Bile acids (BAs), synthesized from cholesterol in the liver are essential signal molecules, modulate lipids metabolism. BA receptors are currently being considered potential therapeutic targets for NAFLD [17]. G-protein-coupled bile acid receptor 1 (TGR5) shows potentials in the browning of white adipose tissues in NASH animal models [18]. BAs can be classified as unconjugated or taurine- or glycine-conjugated BAs—the unconjugated BAs are more toxic [19]. 

The traditional diets of Chinese residents mainly include plant-based foods, with a high intake of cereals, tubers, and vegetables. About 70% and 67% of food calories and proteins, respectively, come from grains [20]. Soybean oil and lard are the most consumed vegetable-derived oil and animal-derived fat, respectively, in China. In Chinese traditional dietary patterns, lard and soybean oil were mixed during cooking. However, the consumption of lard has decreased by nearly 50% over the past 20 years because saturated fatty acid (SFA)—a significant content in lard—was associated with cardiovascular diseases [21,22]. SFAs are regarded as a “killer”, because they have been associated with chronic metabolic diseases since the 1950s [22]. However, in recent years, numerous randomized, double-blind controlled trials, and meta-analysis studies have found that compared with unsaturated fatty acids, intake of SFAs shows no significant difference in cardiovascular disease and obesity risks [23,24]. Previously, we established that a lard and soybean oil mixture was found to be beneficial for lipids metabolism in medium-fat diets [25]. In the present study, we explored the effectiveness of a lard and soybean oil mixture (an LFHC diet) in alleviating NAFLD. Further mechanisms were probed using RNA Sequencing (RNA-seq) and liquid chromatography–mass spectrometry (LC–MS).

## 2. Materials and Methods

### 2.1. Treatment of the Mice 

A total of 90 6-week-old male C57BL/6 J mice were obtained from the Hunan Slake Jingda Experimental Animals Co., Ltd. (Changsha, China). This study was performed in strict accordance with the European Community (Directive 2010/63/EU) for the care and use of laboratory animals. The Hunan Agricultural University, China (No. 43321543) Ethics Committee approved the animal experimental protocol used for this work. Efforts were aimed at minimizing animal suffering and stress. They were housed in individual cages (3 per cage) in a clean room, with a controlled temperature of 22 ± 2 °C, humidity of 65% ± 5%, and light control day and night. All the mice received ad libitum access to tap water and food. After a week of acclimatization, mice were separated into groups and fed with apportioned experimental foods for 16 weeks, respectively.

The mice were fed 1 of the following diets: (1) a lard diet with 10% calories from fat, 75.9% calories from carbohydrate (L1); (2) a soybean oil diet with 10% calories from fat, 75.9% calories from carbohydrate (S1); (3) mixture of lard and soybean oil with 10% calories from fat, 75.9% calories from carbohydrate (LS1); (4) lard diet with 15.5% calories from fat, 72.8% calories from carbohydrate (L2); (5) soybean oil diet with 15.5% calories from fat (S2), 72.8% calories from carbohydrate (L2); (6) mixture of lard and soybean oil with 15.5% calories from fat, 72.8% calories from carbohydrate (L2). Main ingredient compositions of the diets are listed in Table S1. All diets were prepared by trophic Animal Feed High-Tech Co., Ltd. (Nantong, China). The lard was purchased from Tang Ren Shen Co., Ltd. (Hunan, China), the Fu Lin Men one grade soybean oil was procured from COFCO Co., Ltd. (Beijing, China). Fatty acids compositions were tested and are shown in Table S2. Body weight and food intake were monitored weekly.

### 2.2. Sample Collection from the Mice

Before blood collection, each mouse was weighed and labeled, and a 10% chloral hydrate intraperitoneal injection of 4 mL/kg was performed. The blood was left overnight to precipitate in a refrigerator at 4 °C. It was then centrifuged at 3900× *g* at 4 °C for 10 min, and the upper serum was obtained for subsequent testing. The dissected liver was weighed, and the left lobe fixed in neutral formaldehyde—this was used for liver oil red staining and hematoxylin–eosin (HE) staining. The samples were divided into 6 parts and frozen at −80 °C for subsequent detection. Epididymal and perirenal white adipose tissues were collected, weighed, and body fat rates were calculated. Liver index = liver weight (g)/body weight (g) × 100%; Body fat rate = [epididymal white adipose tissue weight (g) + perirenal white adipose tissue weight (g)]/body weight (g) × 100%.

### 2.3. Biochemical Parameters of the Liver and Blood Samples

The SINNOWA biochemical analyzer was used for direct detection of the following: serum alanine aminotransferase (ALT); alkaline phosphatase (ALP); total triglyceride (TG); total cholesterol (TC); aspartate aminotransferase (AST); high-density lipoprotein cholesterol (HDL-C); low-density lipoprotein cholesterol (LDL-C). Serum FFAs, liver FFAs, and liver TG detection kits were procured from Nan Jing Jian Cheng Biological Engineering Co., Ltd. (Nanjing, China).

### 2.4. Histological Analysis

Before histological analysis of the liver, liver specimens were paraffin-embedded, sections were 5 mm thick, and HE staining was performed using standard methods. The liver was also subjected to oil red O (American) staining for 20 min followed by counter-staining with hematoxylin for 1 min. Color areas were evaluated by optical Olympus microscope (Tokyo, Japan) with an enlarged 50× and 400× of oil red O stain. HE staining was observed under 200×.

### 2.5. RNA-Seq

The total RNA from the liver was quantified using the Trizol total RNA Exaction Kit. The input material for RNA sample preparation was 1 μg RNA from each sample. The NEBNext Ultra^TM^ RNA Library Prep Kit for Illumina (NEB) was used to prepare sequencing libraries, as instructed by the manufacturer. First, processing of raw reads in the fastq format was performed via in-house perl scripts. HISAT2 v2.0.5 [26] software was selected to align the filtered sequence and count the reads numbers mapped to each gene. All steps were finished by Novogene Technology Co., Ltd. (Beijing, China).

Differential expression analysis was performed using DESeq R package [27]. The *p*-value results were adjusted by the Benjamini and Hochberg approach for controlling the false discovery rate. Enrichments of differentially expressed genes (DEGs) in the Kyoto Encyclopedia of Genes and Genomes (KEGG) pathways were evaluated using the clusterProfiler 3.4.4 software.

### 2.6. Reverse Transcription Quantitative PCR (RT-qPCR) 

Validation of some differentially expressed genes (DEGs) that had been revealed by RNA-Seq was performed by RT-qPCR using sequenced samples. Briefly, PrimeScript RT reagent Kit (Takara Bio Inc., Japan) was used to reverse transcribe the mRNA into cDNA. The sequences of primers are shown in Table S3. RT-qPCR was conducted using the SYBR Premix Ex Taq IIKit (Takara Bio Inc., Kusatsu, Japan) on the StepOne Real-Time PCR System (Applied Biosystems, Waltham, MA, USA). Normalization of relative CT amounts were performed to β-actin expressions.

### 2.7. Enzyme-Linked Immunosorbent Assay (ELISA) Test

Liver fatty acid-binding protein 2 (FABP2), interleukin 1α (IL-1α), IL-1β, cytochrome P450 family 7 subfamilies A member 1 (CYP7A1), cytochrome P450 family 7 subfamilies B member 1 (CYP7B1), solute carrier family 6 member 6 (SLC6A6), solute carrier family 6 member 9 (SLC6A9), and adipose tissue TGR5 were evaluated via an ELISA assay kit purchased from Elabscience Biotechnology Co., Ltd. (Wuhan, China), and were measured according to the manufacturer’s instructions.

### 2.8. Targeted BAs Quantitative Analysis 

About 50 mg of liver samples were supplemented with 400 μL mixture (methanol: water = 4:1). An LC-ESI-MS/MS system (UHPLC-QE, Thermo Q Exactive) with a Waters BEH C18 Liquid chromatography column (100 × 2.1 mm, 1.7 μm) was used to detect the extracted samples. Automatic identification and integration of ion fragments, as well as manual inspection, were performed by the Xcalibur quantitative software (Thermo, Waltham, MA, USA). With the analytes’ mass spectrum peak area as the ordinate and its concentration as the Abscissa, a linear regression standard curve was established. The analytes’ peak area was substituted into a linear equation to calculate the concentration. SCFA (μg/mg) = (C × V)/M. Note: C denotes GC-MS-determined concentration, V is the volume of constant volume, while M denotes sample weight at extraction time, per unit mg.

### 2.9. Statistical Analysis

Data were expressed as mean ± standard error of the mean (SEM). The distribution of data conforms to normality before statistical analysis except for those with additional instructions. Then, the one-way analysis of variance (ANOVA) was used to analyze statistical differences between the mean values of treatment groups, and the least significant difference or tamhane’s t2; significance was set at *p* < 0.05. The GraphPad Prism V. 7 (Graph Pad Software, San Diego, CA, USA) was used for analyses.

## 3. Results

### 3.1. Effects of Lard and Soybean Oil Mixture on Body Weight and Body Fat Accumulation

**Figure 1:**
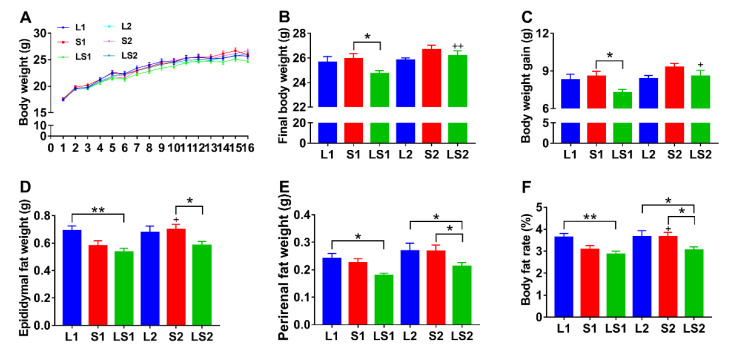
Impacts of various oil/fat diets on (**A**) body weight over 16 weeks, (**B**) final body weight, (**C**) body weight gain, (**D**) epididymal fat weight, (**E**) perirenal fat weight, (**F**) body fat rate. “*” denotes marked differences at *p* < 0.05; “**” denotes significant differences at *p* < 0.01; “+” shows marked differences at *p* < 0.05 relative to the same oil type with different energy level; “++” represents significant differences at *p* < 0.01 relative to the same oil type with different energy level.

The body weights of the mice in the different groups increased throughout the experimental period (Figure 1A). The terminal body weight and weight gain of LS1 mice were low, relative to those of L1 (*p* > 0.05) and S1 (*p* < 0.05) mice; the final body weight and weight gain of LS2 mice were low, relative to those of S2 mice; however, there was no significant difference amongst those of L2, S2, and LS2 mice (Figure 1B,C). LS1 mice epididymal and perirenal fat weight were lower than those of S1 (*p* > 0.05) and L1 (*p* < 0.05) mice. LS2 mice epididymal and perirenal fat weights were markedly lower than those of S2 mice (*p* < 0.05). The LS2 mice perirenal fat weight was also lower than that of L2 mice (*p* < 0.05) (Figure 1D,E). The body fat rate of LS1 mice was significantly lower than that of L1 mice (*p* < 0.01), while LS2 mice body fat rate was markedly lower than those of L2 (*p* < 0.05) and S2 (*p* < 0.05) mice (Figure 1F). The terminal body weight and weight gain of LS2 mice were higher than that of LS1 mice (*p* < 0.05) (Figure 1B,C). Epididymal fat weight and body fat rate of S2 mice were higher than S1 mice (*p* < 0.05) (Figure 1D,F). These findings illustrated that the lard and soybean oil mixture could not substantially decrease body weight, but robustly decreased body fat accumulation.

### 3.2. Effects of Lard and Soybean Oil Mixture on Food Intake

**Figure 2:**
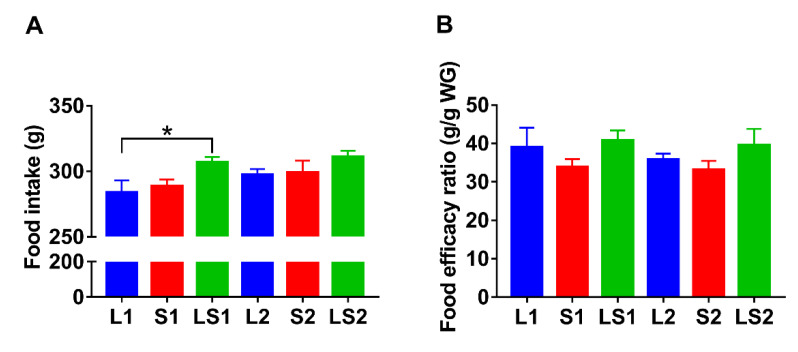
Impacts of various oil/fat diets on (**A**) food intake, (**B**) food efficacy ratio, during a period of 16-week intervention. “*”represents significant differences at *p* < 0.05.

LS1 mice showed a higher amount of food intake than L1 (*p* < 0.05) and S1 (*p* > 0.05) mice. The food intake of LS2 mice was also higher than that of L2 (*p* > 0.05) and S2 (*p* > 0.05) mice (Figure 2A). The food efficacy ratios of S1 and S2 mice were lower than those of the other four groups, but there were no significant differences between the groups (Figure 2B). The food intake results showed that food intake was not the critical factor leading to lowering fat accumulation in mice fed with the lard and soybean oil mixture.

### 3.3. Effects of Lard and Soybean Oil Mixture on Serum Lipids Level

**Figure 3:**
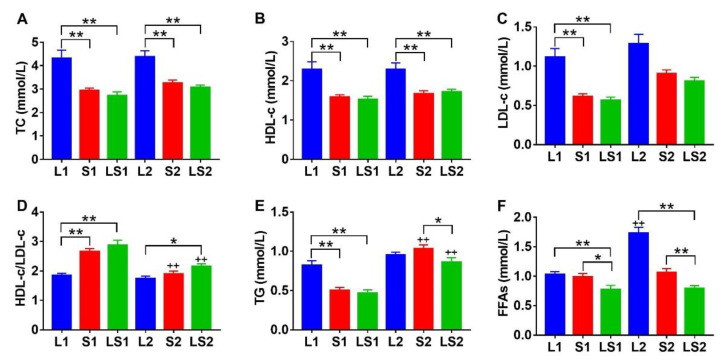
Impacts of various oil/fat diets on serum (**A**) total cholesterol (TC), (**B**) high density lipoprotein cholesterol (HDL-c), (**C**) low density lipoprotein cholesterol (LDL-c), (**D**) ratio of HDL-c/LDL-c, (**E**) triglyceride (TG), (**F**) free fatty acids (FFAs). LDL-c was analyzed using Kruskal–Wallis, significance was determined by two-tailed unpaired *t* test, *p* value was adjusted using Bonferroni. “*” denotes marked differences at *p* < 0.05; “**” denotes marked differences at *p* < 0.01; “++” represented significant differences at *p* < 0.01 relative to the same oil type with different energy level.

The serum TC, HDL-C, and LDL-C concentrations in L1 mice were markedly elevated compared with those of S1 (*p* < 0.01) and LS1 (*p* < 0.01) mice. The serum TC and HDL-C concentrations of L2 mice were markedly high relative to those of S2 (*p* < 0.01) and LS2 (*p* < 0.01) mice; LDL-C centration of L2 mice also higher than S2 (*p* > 0.05) and LS2 (*p* > 0.05) mice (Figure 3A–C). L1 mice HDL-C/LDL-C ratio was low relative to those of L1 (*p* < 0.01) and S1 (*p* < 0.01) mice, L2 mice HDL-C/LDL-C ratio was also high, relative to those of S2 (*p* < 0.05) and LS2 (*p* > 0.05) mice, while S2 and LS2 mice HDL-C/LDL-C ratios were significantly lower than those of S1 (*p* < 0.01) and LS1 (*p* < 0.01) mice (Figure 3D). The L1 mice TG concentration was markedly high, relative to those of S1 (*p* < 0.01) and LS1 (*p* < 0.01) mice, and the LS2 mice TG concentration was significantly lower than those of L2 (*p* > 0.05) and S2 (*p* < 0.05) mice, the S2 and LS2 mice TG concentrations were significantly higher than those of S1 (*p* < 0.01) and LS1 (*p* < 0.01) mice (Figure 3E). The LS1 mice FFAs concentration was significantly lower than those of L1 (*p* < 0.01) and S1 (*p* < 0.05) mice. Similarly, the LS2 mice FFAs concentration was markedly low relative to those of L2 and S2 mice (Figure 3F). These results strongly suggest that the lard diet induced hyperlipidemia; however, the lard and soybean oil mixture improved fatty acids metabolism and reduced FFAs accumulation.

### 3.4. Effects of Lard and Soybean Oil Mixture on Liver Function

**Figure 4:**
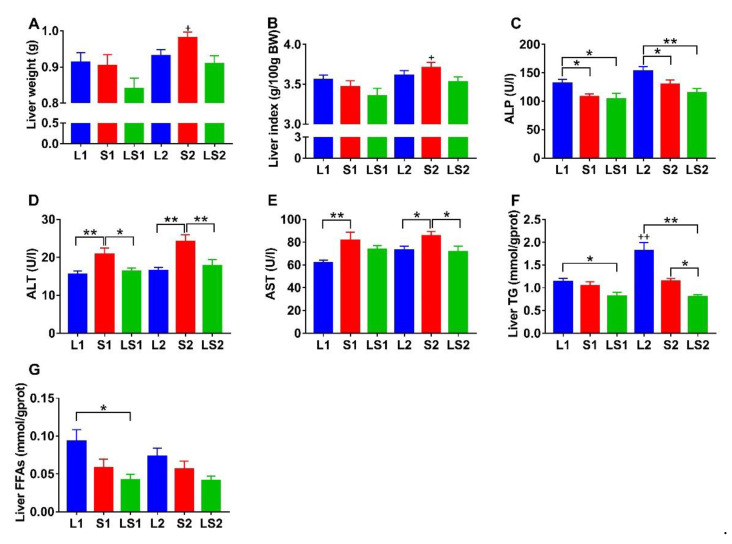
Impacts of various oil/fat diets on (**A**) liver weight, (**B**) liver index, (**C**) alkaline phosphatase (ALP), (**D**) alanine aminotransferase (ALT), (**E**) aspartate aminotransferase (AST), (**F**) liver TG, (**G**) liver FFAs in mice during a period of 16-week intervention. Liver weight and liver FFAs were analyzed using Kruskal–Wallis, significance was determined by two-tailed unpaired *t* test, *p* value was adjusted using Bonferroni. “*” represents significant differences at *p* < 0.05; “**” denotes considerable differences at *p* < 0.01; “+” implies marked variations at *p* < 0.05 relative to the same oil type with different energy level; “++” represents significant differences at *p* < 0.01 relative to the same oil type with different energy level.

The liver weight and liver index of LS1 mice were lower than those of L1 (*p* > 0.05) and S1 (*p* > 0.05) mice. Similarly, the liver weight and liver index of LS2 mice were lower than those of L2 (*p* > 0.05) and S2 (*p* > 0.05) mice (Figure 4A,B). L1 mice displayed substantially higher ALP activity relative to those of S1 (*p* < 0.05) and LS1 (*p* < 0.05) mice, and L2 mice also showed significantly higher ALP activity relative to those of S2 (*p* < 0.05) and LS2 (*p* < 0.01) mice (Figure 4C). The L1 and L2 mice groups’ ALP activities were higher than 100 U/L (critical value of normal range) [28]. The S1 mice ALT and AST activities were substantially higher than that of L1 mice (*p* < 0.01); the S1 mice ALT activity was considerably elevated compared with that of LS1 mice (*p* < 0.05). Similarly, S2 mice ALT and AST activities were considerably higher than those of L2 (*p* < 0.05) and LS2 (*p* < 0.05) mice (Figure 4D,E). All group ALT activity was in the standard range, but their AST activity was higher than 60 U/L (critical value of normal range)—especially for S1 and S2 mice groups. The LS1 mice liver TG content was lower than those of L1 (*p* < 0.05) and S1 (*p* > 0.05) mice; LS2 mice liver TG content was also lower than those of L2 (*p* < 0.01) and S2 (*p* < 0.05) mice; L2 mice liver TG content was markedly higher than that of L1 mice (*p* < 0.01) (Figure 4F). In addition, the L1 mice liver FFAs content was significantly higher than those of S1 (*p* > 0.05) and LS1 (*p* < 0.05) mice. Similarly, L2 mice liver FFAs content was higher than those of S2 (*p* > 0.05) and LS2 (*p* > 0.05) mice (Figure 4G). These results illustrate that the lard and soybean oil mixture potentially has a role in reducing liver injury and lipids accumulation.

### 3.5. Effects of Lard and Soybean Oil Mixture on Liver Pathology

**Figure 5:**
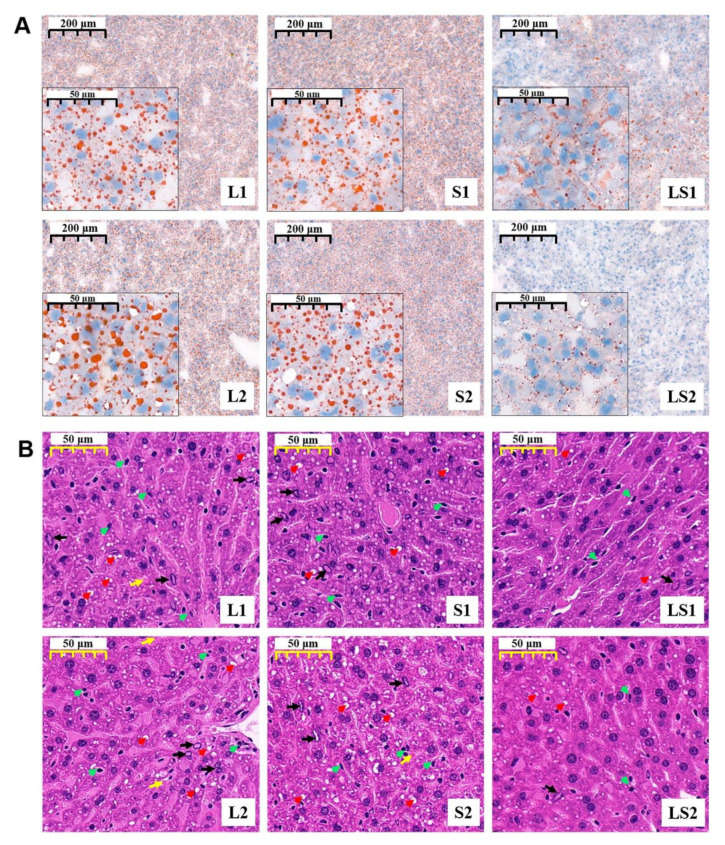
Impacts of various oil/fat diets on liver (**A**) liver oil red staining (magnification 50× and 400×) and (**B**) hematoxylin–eosin staining in mice during a period of 16-week intervention (magnification 200×). Red arrow—circular cavity; green arrow—inflammatory cells; black arrow—edge set of chromatin; yellow arrow—focal infiltration of lymphocytes.

It was observed that there were excess lipids accumulation in L1, S1, L2, and S2 mice liver cells than in LS1 and LS2 (Figure 5A). The stained HE sections show circular cavities (marked with red arrow), edge set of chromatins (marked with black arrow), and inflammatory cells (marked with green arrow) were observed in all groups; however, their occurrence was less frequent in LS1 and LS2 mice liver cells. Additionally, there was no focal infiltration of lymphocytes (marked with yellow arrow) observable in LS1 and LS2 mice liver cells (unlike in the other four groups) (Figure 5B). The HE results reveal that L1, S1, L2, and S2 mice had mild liver steatosis and inflammation, and the lard and soybean oil mixture decreased steatohepatitis.

### 3.6. Transcriptome Analysis and Verification of Differential Expression Genes (DEGs) Involved in Liver Lipids Metabolism

Analyses of transcriptome-wide expressions were conducted by RNA-Seq, resulting in approximately 50 million raw reads, with >91% high quality reads as shown by the Phred quality score (Q score) >30. An Illumina quality score of Q30 denotes an accuracy of 99.9% (Table S4). 

**Figure 6:**
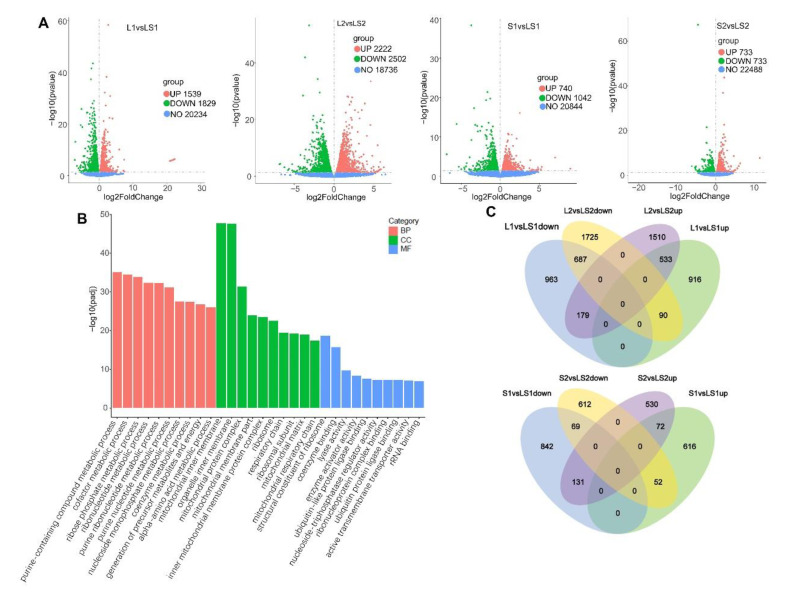
The liver transcriptome analysis of mice fed different oil/fat. (**A**) The volcano of DEGs; (**B**) the GO function classification of DEGs; (**C**) the Venn diagram of DEGs.

Our RNA-seq data needed further analysis. We obtained 1539 elevated and 1829 suppressed genes in total between L1 and LS1 groups; exactly 2222 elevated and 2502 suppressed genes was between L2 and LS2 groups; 740 elevated and 1042 suppressed genes between S1 and LS1 groups; 733 elevated and 733 suppressed genes was between S2 and LS2 group; at a strict log2foldchange > 0 (Figure 6A). Gene ontology (GO) analysis was used to further explore gene functions (Figure 6B). According to GO terms of cellular components (CC), most genes were located at mitochondrion—at the mitochondrial inner membrane, the mitochondrial protein complex, or the mitochondrial respiratory chain. According to molecular function (MF), most genes were involved in “binding”—coenzyme binding, ubiquitin-like protein ligase binding, and ribonucleoprotein complex binding. The GO terms for molecular function showed that most genes participated in “metabolic processes”, such as purine-containing compound metabolic process, generation of precursor metabolites and energy, and alpha-amino acid metabolic process. The Venn diagrams of DEGs accurately displayed specific genes that led to phenotypic difference(s). The Venn diagram displayed the following: 687 overlapped downregulated genes between groups—L1 vs. LS1 group, L2 vs. LS2 group; 533 overlapped upregulated genes between groups—L1 vs. LS1 group, L2 vs. LS2 group; 69 overlapped downregulated genes between groups—S1 vs. LS1 group, S2 vs. LS2 group; 72 overlapped upregulated genes between groups—S1 vs. LS1 group, S2 vs. LS2 group (Figure 6C).

**Figure 7:**
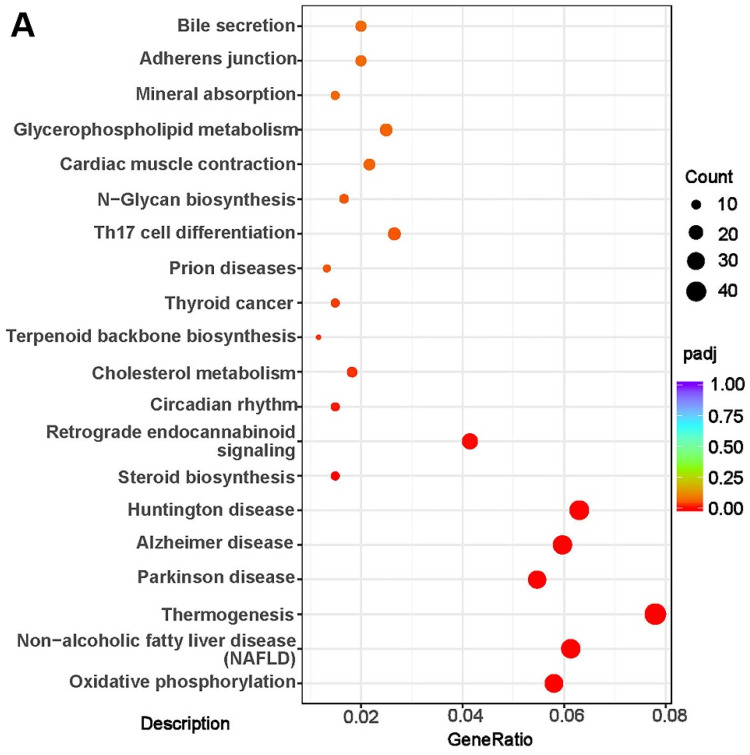

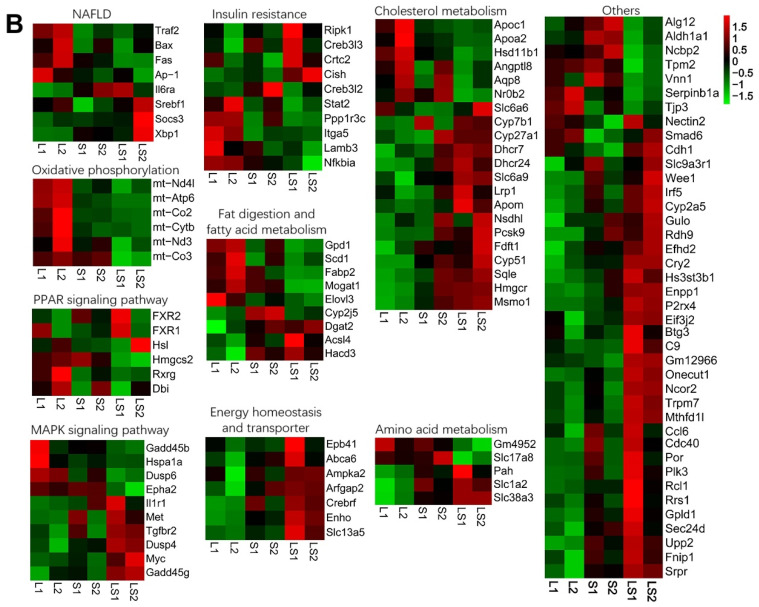
(**A**) Enrichment analysis of differentially expressed genes by KEGG pathway. (**B**) Heatmap of differentially expressed genes.

**Figure 8:**
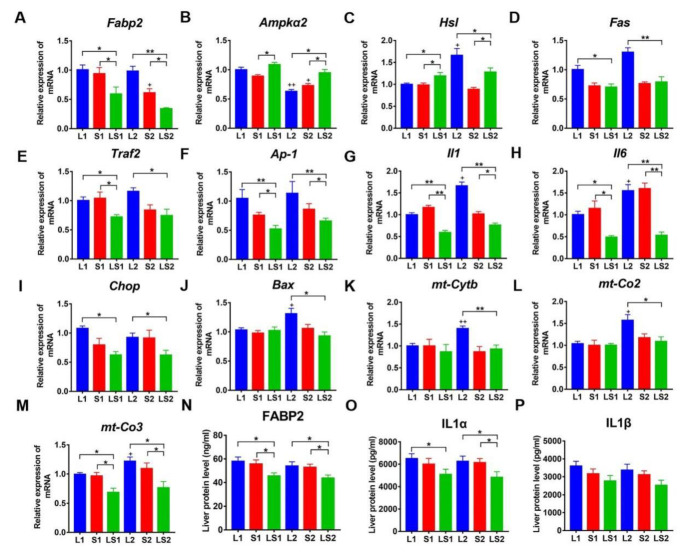
Verified differentially expressed genes of *Fabp2*, *Ampkα2*, *Hsl, Fas, Traf2*, *Ap-1*, *Il1*, *Il6*, *Chop*, *Bax*, mt-*Cytb*, *mt-Co2,* and *mt-Co3* (**A**–**M**, respectively); protein levels of FABP2, IL-1α, and IL-1β (**N**–**P**, respectively). “*” represents significant differences at *p* < 0.05; “**” denotes considerable differences at *p* < 0.01; “+” shows significant variations at *p* < 0.05 relative to the same oil type with different energy level; “++” represents significant differences at *p* < 0.01 relative to the same oil type with different energy level.

The KEGG pathway analysis was performed for 603 DEGs (758 DEGs were overlapped): 37 genes were classified into NAFLD pathway-genes involved in oxidative phosphorylation related reactions, including cytochrome c oxidase III (mt-Co3), nicotinamide adenine dinucleotide (NADH) dehydrogenase 3 (*mt-Nd3*), cytochrome c oxidase II (*mt-Co2*), NADH dehydrogenase 4l (*mt-Nd4l*), cytochrome b (*mt-Cytb*), and adenosine triphosphate synthase 6 (mt-Atp6), which were downregulated in mice fed with mixed oil. Other genes involved with the NAFLD pathway, such as tumor necrosis factor (TNF) receptor superfamily member 6 (Fas), TNF receptor-associated factor 2 (Traf2), activator protein 1 (Ap-1), DNA-damage inducible transcript 3 (*Chop*), and B cell lymphoma 2-associated X protein (*Bax*) were also downregulated in mice fed with mixed oil. The expression of these DEGs was verified by RT-qPCR (Figure 7A,B and Figure 8). Other genes related to fatty acids metabolism, such as stearoyl-coenzyme a desaturase 1 (*Scd1*), *Fabp2*, and retinoid X receptor γ (*Rxr*γ) were all downregulated in mice fed with mixed oil. The levels of FABP2 were verified by RT-qPCR and ELISA (Figure 8). Adenosine 5′-monophosphate-activated protein kinase α2 (*Ampα2*) and *Hsl* genes were upregulated in mice fed with mixed oil, and were verified by RT-qPCR (Figure 8B,C). We also verified inflammatory genes and proteins; results showed IL-6, IL-1α, and IL-1β expressions were downregulated in mice fed with mixed oil (Figure 8). Additionally, we observed 9 steroid biosynthesis-classified genes, 11 cholesterol metabolism genes, and 12 bile secretion genes. Recent studies show that bile acids and cholesterol metabolites are involved in lipids metabolism regulation; based on the above evidence, we tested liver bile acids by LC–MS. 

### 3.7. Effects of Lard and Soybean Oil Mixture on Liver BAs

**Figure 9:**
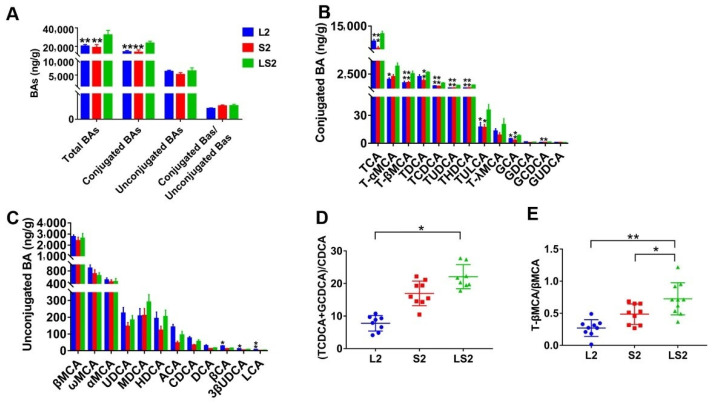
Impacts of various oil/fat diets on liver bile aids. (**A**) Total BAs; (**B**) conjugated BAs; (**C**) unconjugated BAs; (**D**) ratio of (TCDCA+GCDCA) and CDCA; (**E**) ratio of T-βMCA and βMCA. Ratio of (TCDCA+GCDCA) and CDCA was analyzed using Kruskal–Wallis, significance was determined by two-tailed unpaired *t* test, *p* value was adjusted using Bonferroni. “*” denotes considerable differences at *p* < 0.05; “**” shows marked differences at *p* < 0.01.

BAs in mice liver were tested, the results revealed that the total BAs content of LS2 mice were significantly elevated when compared with the other two groups (*p* < 0.01), the elevated BAs were primarily conjugated (as opposed to being unconjugated) (Figure 9A). The separate analysis of all conjugated and unconjugated BAs showed that tauro-conjugated BAs of LS2 mice were significantly elevated; tauro-cholic acid (TCA), tauro-β muricholic acid (T-βMCA), tauro-chenodeoxycholic acid (TCDCA), tauro-ursodeoxycholic acid (TUDCA), tauro-hyodeoxycholic acid (THDCA), tauro-lithocholic acid (TULCA), the glycine-conjugated BAs, glycol-cholic acid (GCA), and glycol-chenodeoxycholic acid (GCDCA) were significantly increased in mice fed with mixed oil (*p* < 0.05 or *p* < 0.01) (Figure 9B). There was no significant difference between the unconjugated mice BAs amongst the groups; except for 3β-Cholic acid (βCA), 3β-Ursodeoxycholic acid (3β-UDCA), and lithocholic acid (LCA) of L2 mice, which were markedly higher than those of LS2 mice (*p* < 0.05 or *p* < 0.01) (Figure 9C). In addition, we calculated (TCDCA+GCDCA)/CDCA and T-βMCA/βMCA, and the results showed that L2 mice had the lowest values, while those of LS2 mice were markedly higher than those of L2 (*p* < 0.05 or *p* < 0.01) mice and were higher than those of S2 mice (*p* > 0.05 or *p* < 0.05) (Figure 9D,E). These bile acids results showed that the lard and soybean oil mixture elevated conjugated BAs liver content.

### 3.8. Effects of Lard and Soybean Oil Mixture on Proteins Related to Bile Acids

**Figure 10:**
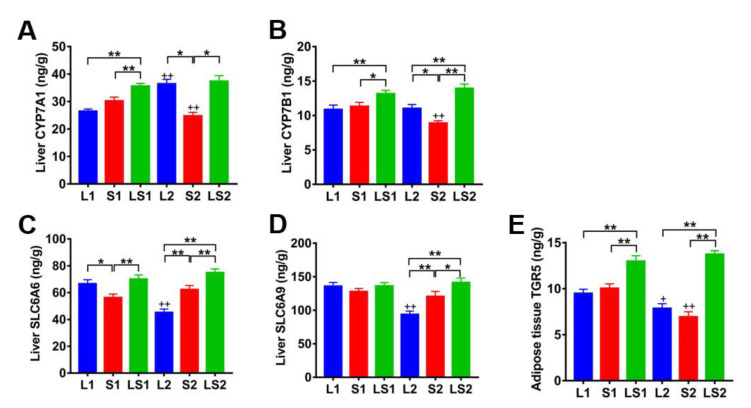
Impacts of various oil/fat diets on proteins related to bile aids. (**A**) Expression of CYP7A1 in liver; (**B**) expression of CYP7B1 in liver; (**C**) levels of SLC6A6 in liver; (**D**) levels of SLC6A9 in liver; (**E**) levels of TGR5 in adipose tissue. “*” represents significant differences at *p* < 0.05; “**” represents significant differences at *p* < 0.01; “+” represents significant differences at *p* < 0.05 relative to the same oil type with different energy level; “++” represents significant differences at *p* < 0.01 relative to the same oil type with different energy level.

The LC–MS results show that mixed oil benefits are closely associated with the BAs and RNA-seq results, which indicates that cholesterol catabolic, transport of taurine, and glycine-related proteins participated in bile acid signal transmission. Therefore, we tested CYP7A1, CYP7B1, SLC6A6, and SLC6A9 liver contents, as well as the content of TGR5 in adipose tissue by ELISA. LS1 mice CYP7A1 and CYP7B1 content were elevated relative to those of L1 (*p* < 0.01) and S1 (*p* < 0.05) mice, LS2 and L2 mice CYP7A1 contents were higher than that of S2 mice (*p* < 0.05), LS2 mice CYP7B1 content was substantially more elevated than those of L2 and S2 mice (*p* < 0.01). In contrast, L2 mice CYP7B1 content was considerably high relative to that of S2 mice (*p* < 0.05), S2 mice CYP7A1 and CYP7B1 contents were markedly suppressed relative to those of S1 mice (*p* < 0.01), and L2 mice CYP7A1 content was significantly higher relative to that of L1 mice (*p* < 0.01) (Figure 10A,B). S1 mice SLC6A6 content was low when compared with those of L1 (*p* < 0.05) and LS1 (*p* < 0.01) mice, LS2 mice SLC6A6 and SLC6A9 contents were considerably higher than those of L2 (*p* < 0.01) and S2 (*p* < 0.05) mice, S2 mice SLC6A6 and SLC6A9 contents were also markedly higher relative to those of L2 mice (*p* < 0.01), and L2 mice SLC6A6 and SLC6A9 contents were substantively lower than that of L1 mice (*p* < 0.01) (Figure 10C,D). LS1 mice TGR5 content was substantially higher than those of L1 (*p* < 0.01) and S1 (*p* < 0.01) mice. Similarly, LS2 mice TGR5 content was substantially higher than those of L2 (*p* < 0.01) and S2 (*p* < 0.01) mice, and L2 and S2 mice TGR5 content were markedly lower than those of L1 (*p* < 0.05) and S1 (*p* < 0.01) mice, respectively (Figure 10E). These results illustrate that the lard and soybean oil mixture promoted cholesterol metabolization to bile acids by enhancing CYP7A1 and CYP7B1 expressions, and the lard and soybean oil mixture advanced taurine and glycine transport by evaluating SLC6A6 and SLC6A9 expressions; thus, the mixture promoted the conjugation of BAs with taurine and glycine, increased conjugated BAs’ liver content, and the conjugated BAs enhanced TGR5 expression.

## 4. Discussion

High-fat diets induce NAFLD in models, and they elicit substantial body weight gain within 6 weeks [29]. A few LFHC diets can also induce NAFLD. Tessitore A et al. [9] proved that long-term LFHC diet can induce NAFLD; moreover, Pompili S et al. [8] found that an LFHC diet was equally as effective as HFD in developing NAFLD in mice. However, LFHC diet-induced NAFLD does not lead to the obese condition; this outcome makes all the difference as the characteristic of NAFLD is similar to patients with “lean NASH” in Asia [7]. In our present study, the LFHC diet did not induce obesity. During the experimental period (16 weeks), mice body weight increased, on average, by 45%. Additionally, results from Pompili S et al. [8] showed that the proportion of body weight increment did not exceed 70%; however, an HFD induced a 3–4-fold body weight increase within 16 weeks. Body weight and fat accumulation may be closely associated with high-energy diets. Hu S et al. [30] published a work in *Cell Metabolism*, which illustrates that fat macronutrients lead to adiposity—not protein, nor carbohydrate. In addition, it was observed that the body fat rate of mice fed with the lard and soybean oil mixture was lower than those of mice fed with lard (~21%) and soybean oil (~16.7%) separately—illustrating that the lard and soybean oil mixture is beneficial in improved TG metabolism.

NAFLD includes simple liver steatosis and NASH. Our result showed that the lard and soybean oil mixture reduced liver TG and FFAs accumulation and alleviated liver steatosis. Besides, the mixture reduced ALP and AST activities to the standard range and reduced alleviated NASH-induced inflammation. Excess amounts of TG and FFAs, especially FFAs that predispose to lipotoxicity, promote NASH development [31]. The lard and soybean oil mixture suppressed TG and FFAs accumulation in serum and lipids in liver cells compared with lard and soybean oil separately. Some evidence shows that SFA intake increases liver lipids accumulation in rodents [32,33,34], and lard diets, rich in SFA, and soybean oil diets, rich in polyunsaturated fatty acid, are both harmful to organisms [35,36,37]. Henkel J et al. [38] observed that the consumption of soybean oil with cholesterol led to more severe NASH than lard with a cholesterol diet. In our present study, the lard diet induced more serum and liver lipid accumulation, which may be closely related to the high FFAs released from lard [39]. Additionally, serum LDL-C concentration in mice fed with lard was substantially higher than those fed with soybean oil and the lard and soybean oil mixture. The HDL-C/LDL-C ratio in mice fed with lard was significantly lower than those fed with soybean oil and lard and soybean oil mixture. Numerous studies have reported that lard, rich in SFA, increased serum TC and LDL-C content, especially [40,41]. Hayes KC et al. [42] found that serum HDL-C increased when serum LDL-C reduced with SFA intake, consistent with our present results. 

The RNA-seq results showed that lard and soybean oil mixture downregulated expressions of *Fabp2*, *Fas*, *Ap-1*, *Traf2*, *mt-Cytb*, *mt-Co2*, *mt-Co3*, *IL-1α*, *IL-1β*, and *IL-6*, and upregulated *Ampk*α2 and *Hsl* expressions. *Fabps* displayed a high affinity for binding to long chain (>14 C) fatty acids [43,44]. FABPs are present in high abundance (1~5%) in the cytosol of various tissues, such as liver, intestinal, kidney, and adipose tissue [45]. FABP2, also called IFABP, is highly expressed in the intestine: publications also found they are expressed in the liver [46,47]. *Fabp2*^-/-^ mice displayed a lean phenotype [48]. It was reported that FFAs transferred from *Fabp2* are mediated via direct collisional interactions between the *Fabp2* and membranes [49,50]. The charge–charge interactions are considered as a driving force for *Fabp2*-mediated FFAs transfer [45]. *Ampk* plays a significant role in cellular energy balance regulation. Upregulated *Ampk*α2 mRNA is associated with lower hepatic lipid accumulation [51]. The activation of AMPK significantly affects *Hsl* and *Scd1* expressions, both genes are related to fatty acids metabolism [52]. Overall, HSL is the rate-limiting enzyme of TG lipolysis to FFAs [53]. Lower FFAs accumulation in mice fed with the lard and soybean oil mixture may have contributed to the lowered FABP2 expression and higher expressions of *AMPKα2* and *Hsl* expressions than other mice groups. *Fas* is a vital death receptor: it interacts with the Fas ligand, leading to the membrane–proximal scaffolding complex formation that induces caspase recruitment as well as activation and eventually apoptosis induction [54]. It was reported that FFAs could induce *Fas* genes expression [55]. Exposure of hepatocytes to FFAs induces endoplasmic reticulum stress by activating Ire1 binding to the TNFα receptor-associated factor 2 (Traf2). It leads to the upregulation of AP-1 expression and the secretion of proinflammatory cytokines [56]. The generation of cellular ATP via oxidative phosphorylation in the mitochondrial electron transport chain uses various electron transfer reactions. Electron transfer enhances reactive oxygen species (ROS) production, contributing to homeostatic signaling and oxidative stress during pathology [57]. Mitochondria are the most significant source of ROS in NAFLD [58]. Complexes I, II, III, IV, and V participate in oxidative phosphorylation and involve mt-Cytb, mt-Co2, and mt-Co3 [59]. Impairment of the electron transport chain promotes electron leakages and subsequent molecular oxygen activation to form hydrogen peroxide and superoxide. The formation of the “oxides” induces mitochondrial damage and apoptosis [58]. In this study, the lard and soybean oil mixture downregulated Fas, Traf2, Ap-1, mt-Cytb, mt-Co3, mt-Co2, IL-1α, IL-1β, and IL-6 expressions. RNA-seq results revealed that the lard and soybean oil diet mixture reduced FFAs accumulation via downregulating the expression of Fabp2, and alleviating FFAs-induced inflammation. Both appear to involve the downregulation of Fas, and other genes involved in endoplasmic reticulum stress.

**Figure 11:**
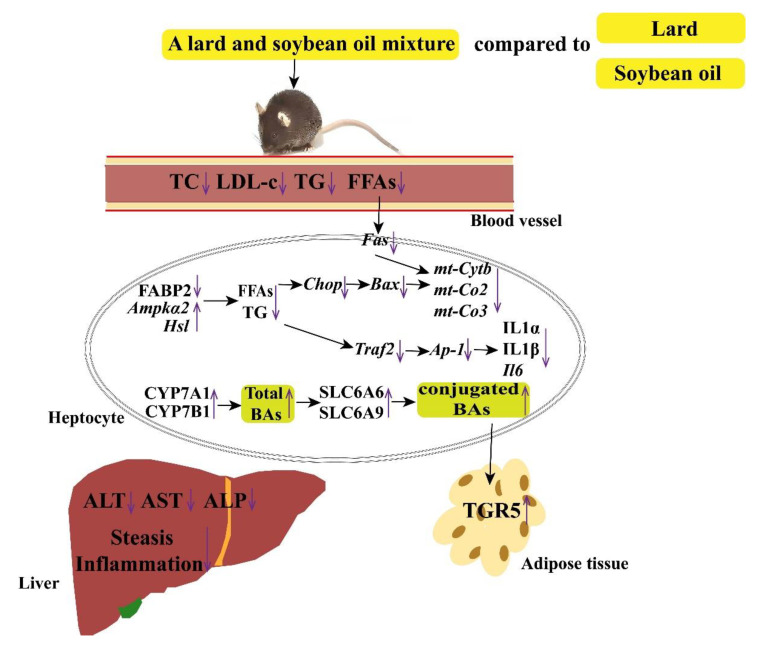
The mechanism of mixture of lard and soybean oil alleviates low-fat–high-carbohydrate diet-induced nonalcoholic fatty liver disease.

BAs are cholesterol metabolites. Primary bile acids, such as CA and CDCA in humans, as well as αMCA, CA, UDCA, and βMCA in mice, are synthesized in the liver through various enzymatic modifications via the classical or the alternative pathways, respectively [60]. The classical pathway is induced by 7a-hydroxylation of cholesterol under the action of the CYP7A1 enzyme [61]. The rate-limiting enzyme (CYP7A1) determines the number of produced bile acids. Sterol-27-hydroxylase initiated the alternative (or acidic) pathway. The formed 27-hydroxycholesterol is then hydroxylated by CYP7B1. Predominantly, the alternative pathway generates CDCA, while the classical pathway generates CA and CDCA. The ratio of these two primary bile acids is regulated by the sterol 12a-hydroxylase (CYP8B1), which is necessary for CA synthesis [62]. To enhance solubility, the newly synthesized bile acids are conjugated with hydrophilic molecules, such as taurine or glycine [63]. SLC6A9 and SLC6A6 genes encode a multi-pass membrane protein that transports glycine or taurine, respectively [64,65]. It was reported that the proportion of conjugated bile acid is positively connected with lipids accumulation in mice [66]. Our results illustrated that lard and soybean oil mixture promoted bile acid synthesis upregulated CYP7A1 and CYP7B1 expressions, and facilitated taurine or glycine conjugation with bile acids. Apart from their “classical” roles in bile generation and intestinal dietary lipid absorption, various vital physiological bile acid roles have been reported. Bile acids exert hormone-like roles in regulation of lipid and glucose, as well as energy metabolism [63]. The roles of bile acids are mediated through activation of various receptors: farnesoid X receptor and TGR5 have been recognized as drug targets for NAFLD treatment [17]. This study confirms that TGR5 signals control glucose homeostasis by increasing energy expenditure in brown adipose tissue [67]. Some evidence shows that TCA, TαMCA, TβMCA, and TCDCA could activate TGR5 in adipose tissues [68,69,70]. Our results also show that conjugated BAs were facilitated by the lard and soybean oil mixture and alleviated NAFLD via TGR5. The benefit and mechanism of a lard and soybean oil mixture was concluded in Figure 11.

## 5. Conclusions

In conclusion, our results demonstrate that a diet with lard and soybean oil mixture could alleviate LFHC diet-induced NAFLD by regulating mice genes and bile acids profiles. The lard and soybean oil mixture alleviated NAFLD by downregulating Fabp2, Fas, Traf2, Ap-1, mt-Cytb, Il-6, and Il-1 genes, upregulating Ampkα2 and Hsl genes, and promoting conjugated BAs and BAs signal receptor TGR5 protein. This finding is essential for the prevention of lean NAFLD in Asians.

## Supplementary Materials

The following supporting information can be downloaded at: https://www.mdpi.com/article/10.3390/nu14030560/s1, Table S1: Composition of diets; Table S2: Fatty acid composition of fats/oils; Table S3: Primer sequences used for quantitative real-time PCR; Table S4: Summary of RNA sequencing (RNA-Seq) data with quality trimming.
