# Supplementary material for: A Lard and Soybean Oil Mixture Alleviates Low-Fat–High-Carbohydrate Diet-Induced Nonalcoholic Fatty Liver Disease in Mice
Source: Nutrients. 2022 Jan 27;14(3):560. doi: 10.3390/nu14030560 (PMC8840387; doi:10.3390/nu14030560)
Supplement: Supplementary file 1 [file nutrients-14-00560-s001.zip › nutrients-1518508-supplementary.pdf]

Table S1 Composition of diets

	L1	S1	LS1	L2	S2	LS2
Casein (g)	139	139	139	118.9	118.9	118.9
Corn starch (g)	467.3	467.3	467.3	450.6	450.6	450.6
Dextrin	154.9	154.9	154.9	160.1	160.1	160.1
Sucrose	99.9	99.9	99.9	103.3	103.3	103.3
Lard	39.6	0	18.22	63.4	0	29.16
Soybean oil	0	39.6	21.38	0	63.4	34.24
Cellulose	50	50	50	51.7	51.7	51.7
Mineral	35	35	35	36.2	36.2	36.2
Vitamin	10	10	10	10.3	10.3	10.3
L-cystine	1.8	1.8	1.8	1.9	1.9	1.9
Choline chloride	2.5	2.5	2.5	2.6	2.6	2.6
Calcium hydrogen phosphate	0	0	0	1	1	1
TBHQ	0.01	0.01	0.01	0.01	0.01	0.01
Total	1000	1000	1000	1000	1000	1000
Calorie						
Caloric density (Kcal/g)	3.6	3.6	3.6	3.7	3.7	3.7
Protein (%)	14.10	14.10	14.10	11.7	11.7	11.7
Carbohydrate (%)	75.90	75.90	75.90	72.8	72.8	72.8
Fat (%)	10	10	10	15.5	15.5	15.5
Total (%)	100	100	100	100	100	100

Table S2 Fatty acid composition of fats/oils

	Lard	Soybean oil	Mixed oil
Fatty acids	% total fatty		
C4:0			
C6:0			
C8:0			
C10:0	0.08		0.04
C11:0			
C12:0	0.07		0.03
C13:0			
C14:0	1.49	0.08	0.73
C14:1			
C15:0	0.06		0.03
C15:1			
C16:0	28.80	10.80	19.08
C16:1	1.52	0.09	0.75
C17:0	0.83	0.19	0.48
C17:1			
C18:0	20.50	3.94	11.56
C18:1n9t	0.11		0.05
C18:1n9c	33.60	23.80	28.31
C18:2n6t			
C18:2n6c	10.90	53.20	33.74
C20:0	0.27	0.37	0.32
C18:3n6			
C20:1	0.68	0.69	0.69
C18:3n3	0.49	6.25	3.60
C21:0			
C20:2	0.45		0.21
C22:0		0.43	0.23
C20:3n6			
C22:1n9		0.10	0.05
C20:3n3			
C20:4n6	0.22		0.10
C23:0			
C22:2			
C24:0		0.09	0.05
C20:5n3			
C24:1			
C22:6n3			
SFA	52.10	15.90	32.60
MUFA	35.80	24.58	29.74
PUFA	11.61	59.45	37.44
n6/n3	22.69	8.51	9.40

The fatty acid profiles were identified using Aglient Gas Chromatograph 7890A in according to the GB 5009.168-2016, fatty acid test in food. Blank space means the fatty acid was undetectable.

Table S3 Primer sequences used for quantitative real-time PCR

Gene	Primer sequences
β-actin	CATCCGTAAAGACCTCTATGCCAAC
	ATGGAGCCACCGATCCACA
Il1	TGCCACCTTTTGACAGTGATG
	TGATGTGCTGCTGCGAGATT
Il6	TGATGGATGCTACCAAACTGGA
	TGTGACTCCAGCTTATCTCTTGG
Fabp2	GCCTGGACCATTGAGGGAAA
	GCTTGGCCTCAACTCCTTCA
Ap1	ACCACTTGCCCAACAGAT
	TTGATCCGCTCCTGAGACTC
Traf2	TAGCCCTACTGCTGAGCTCA
	GCTACAAGCCTCTGCAGTCA
Fas	TCTGCGATGAAGAGCATGGTT
	GCAGCGAACACAGTGTTCACA
Bax	AGACACCTGAGCTGACCTTGGAGCA
	GCCCATCTTCTTCCAGATGGTGAGC
mt-Cytb	AACCTCCTATCAGCCATCCC
	TTTCGTGGAGGAAGAGGAGG
mt-Co2	ACCGAGTCGTTCTGCCAATA
	ATTTAGTCGGCCTGGGATGG
mt-Co3	ATTAACCCTTGGCCTACTCACC
	GCAGCCTCCTAGATCATGTGT
Hsl	TCTGCATCACTGTGTCCCTT
	AATGGTCCTCTGCCTCTGTC
Ampkα2	ACGATGAGGCTGTGAAGGAA
	CTGATGGAGGACTAGAGGCG

RT-qPCR was performed in a reaction volume of 20 μL per well, containing 2 μL (200 ng) of cDNA, 0.8 μL of 10 μmol/L forward and reverse primers, 10 μL of SYBR Premix Ex Taq II, 0.4 μL of ROX Reference Dye, and 6 μL deionized water. PCR reactions started by an initial denaturing step at 95 °C for 30 s, followed by 40 cycles of 5 s at 95 °C and 30 s at 60 °C. Relative CT amounts were normalized to β-actin expression, and the results are calculated using the $2^{-\triangle\triangle Ct}$ method.

Table S4 Summary of RNA sequencing (RNA-Seq) data with quality trimming

Samples	Raw Reads	Clean Reads	Clean %	Q30	GC_pct
L1 rep1	62936332	61021486	96.96	94.62	48.27
L1 rep2	73991956	72034982	97.36	94.57	48.82
L1 rep3	68901732	65261592	94.72	94.83	48.66
S1 rep1	50946666	48607218	95.41	94.73	48.96
S1 rep2	56352896	55308924	98.15	94.84	48.55
S1 rep3	42573676	41049168	96.42	94.94	48.31
LS1 rep1	58172740	56777980	97.60	95.01	49.8
LS1 rep2	54620960	53498972	97.95	94.71	49.03
LS1 rep3	61959814	58026844	93.65	94.79	47.66
L2 rep1	56079310	52171176	93.03	95.12	48.78
L2 rep2	66635952	60732902	91.14	94	47.73
L2 rep3	63467386	58115416	91.57	95	48.01
S2 rep1	59996518	58513762	97.53	94.93	48.98
S2 rep2	54174430	52807044	97.92	94.08	48.86
S2 rep3	56560614	55382268	97.48	94.91	49.01
LS2 rep1	56805642	54399486	95.35	94.72	48.8
LS2 rep2	58397856	57117216	97.81	94.82	49.34
LS2 rep3	69710540	66471738	95.76	94.65	48.16
